# Conservation, Variability and the Modeling of Active Protein Kinases

**DOI:** 10.1371/journal.pone.0000982

**Published:** 2007-10-03

**Authors:** James D. R. Knight, Bin Qian, David Baker, Rashmi Kothary

**Affiliations:** 1 Molecular Medicine Program, Ottawa Health Research Institute, Ottawa, Ontario, Canada; 2 The University of Ottawa Centre for Neuromuscular Disease, Ottawa, Ontario, Canada; 3 Department of Cellular and Molecular Medicine, University of Ottawa, Ottawa, Ontario, Canada; 4 Department of Biochemistry, University of Washington, Seattle, Washington, United States of America; 5 Department of Medicine, University of Ottawa, Ottawa, Ontario, Canada; National Institute on Aging, United States of America

## Abstract

The human proteome is rich with protein kinases, and this richness has made the kinase of crucial importance in initiating and maintaining cell behavior. Elucidating cell signaling networks and manipulating their components to understand and alter behavior require well designed inhibitors. These inhibitors are needed in culture to cause and study network perturbations, and the same compounds can be used as drugs to treat disease. Understanding the structural biology of protein kinases in detail, including their commonalities, differences and modes of substrate interaction, is necessary for designing high quality inhibitors that will be of true use for cell biology and disease therapy. To this end, we here report on a structural analysis of all available active-conformation protein kinases, discussing residue conservation, the novel features of such conservation, unique properties of atypical kinases and variability in the context of substrate binding. We also demonstrate how this information can be used for structure prediction. Our findings will be of use not only in understanding protein kinase function and evolution, but they highlight the flaws inherent in kinase drug design as commonly practiced and dictate an appropriate strategy for the sophisticated design of specific inhibitors for use in the laboratory and disease therapy.

## Introduction

Protein kinases are the most ubiquitous single family of signaling molecules in the cell, accounting for approximately 2% of the proteins encoded by the human genome [1]. The simple mechanism of attaching an ATP-derived phosphate to a protein involves kinases in every aspect of cell behavior, from apoptosis to survival, proliferation to differentiation, maturation etc. Protein kinases provide a unique opportunity for understanding proteins in general by presenting us with a seeming paradox: wide scale similarity of sequence and structure combined with a diversity of behavioral consequences to their activity. The vast majority of protein kinases have readily detectable sequence similarity, which translates into structure. But even those known protein kinases that show no significant algorithm-detectable similarity at the level of sequence are believed to have very typical structures, as is evidenced by specific examples [2], [3]. As they all have a shared function in transferring the terminal phosphate of ATP to another protein, similarity is understandable. Evidence to date also suggests a common catalytic mechanism (the possible exception may be the integrin-linked kinase [4]), whereby ATP and an active site divalent cation are bound in identical fashions and phosphotransfer is achieved by a shared set of amino acids. Studies in yeast [5], [6] have shown that kinases can be promiscuous, phosphorylating hundreds of proteins, but they also have clear specificities. How is this specificity attained by one family of highly similar proteins? This paradox suggests the perfection of the kinase as an enzyme: a region ideally suited for the common function of catalysis, with another region(s) uniquely modifiable to attain substrate specificity without altering fold, compromising ligand binding or the subsequent reaction mechanism. A thorough understanding of this family of proteins would generate a tremendous knowledge base for discovering and predicting protein interactions, for designing highly specific and potent inhibitors, and, as a consequence of these facts, for understanding the cell and disease.

As protein kinases are the key players in cell signaling, aberrations in their activity have been directly correlated with numerous disease states (for example, breast cancer [7] and chronic myeloid leukemia [8]) and made them potential targets for drug design in many other diseases (for example, Crohn's [9] and cerebral vasospasm [10]). This has made the kinase the drug target of choice [11]. However, there is an inherent flaw in traditional kinase inhibitor design. Almost all inhibitors target the ATP binding pocket based on a simple principle: if ATP cannot be bound, phosphorylation cannot occur. Building a molecule that can occupy this pocket is relatively simple, but since the ATP binding pocket and the regions in its immediate vicinity are the areas of greatest conservation, building a *specific* inhibitor is impossible. The inherent multi-target nature of inhibitors has been demonstrated by Fabian *et al*. [12], where the twenty compounds tested had multi-target coverage with only 23% of the kinome screened. Other ATP binding proteins could very likely display affinities for these compounds as well, making these inhibitors not just multi-kinase but multi-enzyme. In the laboratory, how can the effect of treating cells with such inhibitors be dissected? And when used for disease, what non-intended effects may arise in the targeted cell type or others over the long term? In the hopes of producing specific inhibitors, what is needed is a new approach to kinase drug design, one which logically targets the region of greatest dissimilarity.

True dissimilarity can be known if similarity or conservation is understood in detail. For this, structure-based comparative approaches are needed to fully extract the information hidden in the three-dimensional protein-structure space. Traditional structure-driven alignment studies concentrate on maximizing fold overlap, and for the highly-similar protein kinase family which has a largely conserved fold, this can be a useful approach. But it is not necessarily the correct one, particularly where inhibitor design is concerned. Due to the information available in a three-dimensional space, structures can be aligned in other ways, for example by using geometry independent of connectivity. Fold can be ignored and focus directed upon residues free from their covalent associations. The positioning of side-chains and those functional groups involved in enzymatic catalysis and protein interactions can be directly overlain for studying similarity and variability. This type of alignment, and not that of fold, is of greater relevance for understanding protein interactions and therefore in designing small molecules or peptides to act as inhibitors.

Understanding the similar/conserved and dissimilar/non-conserved aspects of protein kinases allows for effective drug design. In addition, conservational studies will aid especially in structure prediction. There are at least 518 known human protein kinases [1] and deriving crystal structures for them all would involve a great deal of time and effort. As all known protein kinases have similar structures, homology-driven approaches to structure prediction that incorporate knowledge of conservation should prove fertile. Having a reliable predicted structural kinome would be of great practical use.

In this paper we report on a structural analysis of and a modeling approach to active-conformation protein kinases. We describe the variability found between these kinases in terms of fold and amino-acid side-chain positioning. These results were produced using a novel structural alignment algorithm that will also be described. This algorithm superimposes structures independent of fold to maximize side-chain similarity. The result is not only an alignment but a consensus structure that depicts residue conservation as a distribution of amino acids and amino-acid categories. This consensus can be used to guide structure prediction, and we report here on its successful use with Rosetta [13] in predicting the structure of 3-phosphoinositide dependent protein kinase-1 (PDK1) and the atypical protein kinase Rio2.

## Results

### Alignment

**Figure 1:**
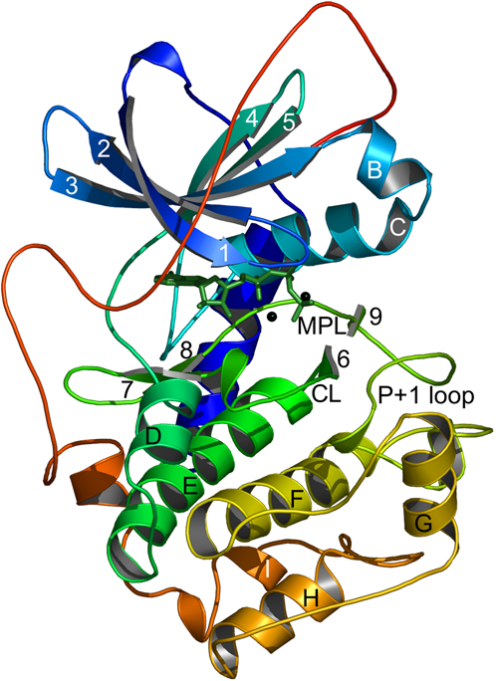
The structure of protein kinase A (PKA). PKA is shown in its active conformation with ATP in green sticks and Mn^2+^ as black spheres. β-strands, helices and loops are labeled as in Knighton *et al*. [45]. The active site is situated between the small and large lobes, located above and below ATP respectively. CL: catalytic loop; MPL: magnesium-positioning loop.

To examine conservation and variability between protein kinases we focused on a group of active-conformation structures. Obviously, it is important to examine like conformations so that any observed variability is in fact real. We defined an active kinase structure as one with ATP or a non-hydrolysable ATP analog, at least one divalent cation (always Mg^2+^ or Mn^2+^), and any necessary phosphorylations. Kinases can be constitutively active or be regulated positively or negatively by phosphorylation, which is ultimately kinase specific. Information regarding the kinase structures used in our analysis can be found in **Table 1**, and an example of an active-conformation protein kinase is shown in **Figure 1**.

**Table 1:** Active-conformation kinase structures.*

Kinase	Full name	Species	PDB code	Pfam domain (residues)
ACK1	activated CDC42 kinase 1	*H. sapiens*	1U54 [46]	Protein tyrosine kinase (126-385)
Akt2	RAC-β serine/threonine-protein kinase	*H. sapiens*	1O6K [25]	Protein kinase (152-409)
CDK2	cell division protein kinase 2	*H. sapiens*	1JST [47]	Protein kinase (4-286)
CK1	casein kinase I	*S. pombe*	1CSN [48]	Protein kinase (12-237)
CK2	casein kinase II subunit α	*Z. mays*	1LP4 [49]	Protein kinase (34-319)
DAPK	death-associated protein kinase	*H. sapiens*	1IG1 [50]	Protein kinase (13-275)
IRK	insulin receptor tyrosine kinase	*H. sapiens*	1IR3 [51]	Protein tyrosine kinase (1023-1290)
MAPK p38γ	mitogen-activated protein kinase p38γ	*H. sapiens*	1CM8 [52]	Protein kinase (27-311)
PhK	phosphorylase kinase	*O. cuniculus*	1PHK [53]	Protein kinase (19-287)
Pim-1	proto-oncogene serine/threonine-protein kinase Pim-1	*H. sapiens*	1XR1 [54]	Protein kinase (129-381)
PKA	protein kinase A	*M. musculus*	1ATP [23]	Protein kinase (43-297)
PknB	probable serine/threonine-protein kinase PknB	*M. tuberculosis*	1MRU [55]	Protein kinase (11-273)
Rio2	Rio2 serine kinase	*A. fulgidus*	1ZAO [56]	Rio1 family (105-275)
Sky1P	SR protein kinase	*S. cerevisiae*	1Q97 [57]	Protein kinase (158-706)†
TAO2	thousand and one amino-acid protein 2	*R. norvegicus*	1U5R [58]	Protein kinase (28-281)
ChaK‡	transient receptor potential channel kinase	*M. musculus*	1IA9 [2]	Alpha kinase (1596-1814)

* The average pair-wise sequence identity between this set as computed by ClustalW using its default parameters is 17%.

† Residues 304-541 constitute a large spacer within the kinase domain [59].

‡ ChaK lacks a divalent cation in its active site and was not used to generate an initial alignment (see Results section).

**Figure 2:**
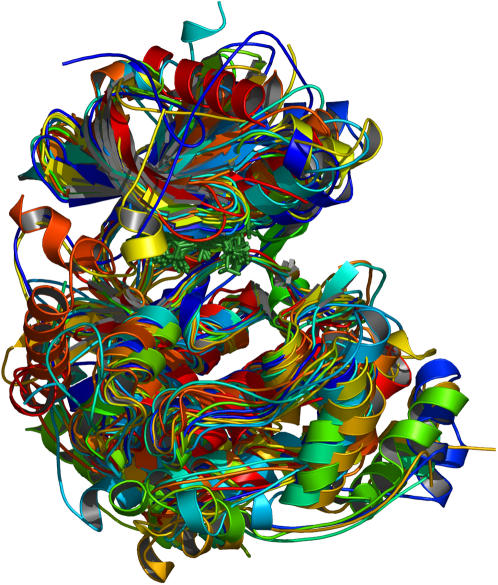
Multiple kinase alignment. The fifteen active-conformation kinase structures listed in Table 1 were aligned using our modified Procrustes approach. Shown in green sticks is the ATP or ATP analog molecule of each structure. Each kinase is colored uniquely.

**Figure 3:**
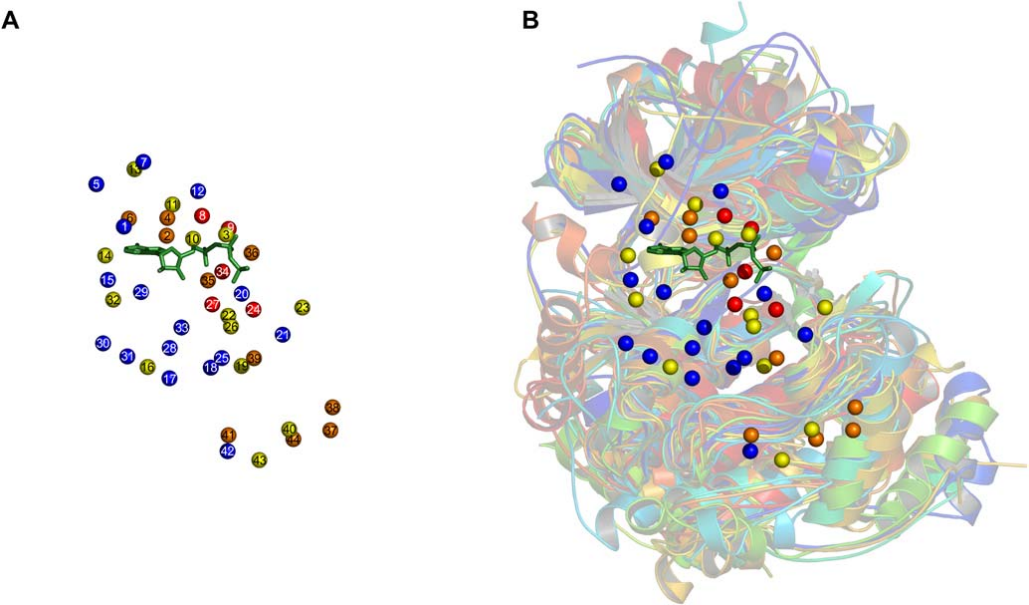
A kinase consensus structure. Each sphere represents a conserved residue. Red indicates full conservation of a particular amino acid in all fifteen kinase structures; orange, conservation in thirteen or fourteen structures; and yellow, conservation in eleven or twelve structures. Blue spheres indicate full conservation of an amino-acid category. The ATP molecule of protein kinase A is shown in green sticks. (A) The consensus structure consisting of the forty-four points listed in Table 2. (B) The consensus structure overlaid on the multiple alignment.

Fifteen kinase structures were aligned using the sequence-order independent algorithm outlined in the Methods section to yield a consensus set of forty-four fully and partially conserved residues. The complete set is listed in **Table 2**. The structural alignment produced is shown in **Figure 2**. From such an image it can be seen that the overall kinase shape is a highly conserved feature. The active site occurs between two lobes: the small lobe above ATP and the large lobe below. Of particular importance for later discussion is the conservation of the substrate-binding groove, located between the catalytic loop, the P+1 loop, helix D, helix F, helix G and helix H. Conserved residues are shown in **Figure 3**, in what we term a consensus structure. This is a distribution of amino acids and amino-acid categories conserved between the protein kinases we have examined. The consensus structure has three principle parts: 1) a region of hydrophobic residues clustered around the adenosine of ATP; 2) an area around the γ-phosphate of ATP – the active site – enclosed primarily by charged residues; and 3) a region in the large lobe, situated below ATP, of both hydrophobic and polar residues. The hydrophobic region around the adenosine creates a binding pocket for ATP. The charged residues in the active site bind and position the γ-phosphate, as well as the divalent cation, and participate in the catalytic mechanism. The conserved residues located in the large lobe serve to stabilize that region, and may play a role in mediating substrate interactions. Only five specific amino acids are fully conserved in all the kinases. These residues play critical parts in positioning ATP, stabilizing the active-conformation and in the catalytic mechanism. These are lysine 8, which interacts with the α- and β- phosphates of ATP, thereby stabilizing it. Glutamic acid 9, which forms a salt bridge with lysine 8 further stabilizing ATP. Aspartic acid 24 is the catalytic base that initiates phosphotransfer by deprotonating the acceptor serine, threonine or tyrosine. Asparagine 27 interacts with a secondary divalent cation, thereby positioning the γ-phosphate of ATP. And the final fully conserved residue is aspartic acid 34, which chelates the primary divalent cation, indirectly positioning ATP at the same time. Although these are the only residues fully conserved in terms of function, location and amino-acid type, there is one other residue with functional conservation but not locational or type, and in other kinases there is variability in the origin of lysine 8 and the type of amino acid fulfilling its role.

**Table 2:** Conserved residues found in the active-conformation kinases listed in Table 1.

	Type	ACK1	Akt2	CDK2	CK1	CK2	DAPK	IRK	p38γ	PhK	Pim-1	PKA	PknB	Rio2	Sky1P	TAO2	Chak*
1	h	L132	L158	I10	I18	V45	L19	L1002	V33	L25	L44	L49	L17	M98	L164	I34	x
2	G	133	159	11	19	46	20	1003	x	26	45	50	18	x	165	35	x
3	G	x	161	13	21	48	22	1005	x	28	x	52	20	x	167	37	1619
4	V	140	166	18	I26l	53	27	1010	41	33	52	57	25	106	172	42	A1624l
5	h	V155	Y178	V30	V38	C65	Y39	V1027	V53	Y45	V64	Y69	V37	C117	V184	V54	Y1643
6	A	156	179	31	39	I66l	40	1028	54	46	65	70	38	V118l	185	55	I1644l
7	h	V157	M180	L32	I40	I67	A41	V1029	I55	V47	I66	M71	V39	V119	M186	I56	I1645
8	K	158	181	33	41	68	42	1030	56	48	67	72	40	120	187	57	1646/R1622b
9	E	177	200	51	55	81	64	1047	74	73	89	91	59	154	202	76	1672
10	L	M181h	204	55	Y59h	85	68	M1051h	78	77	93	95	A63l	158	206	80	x
11	L	192	215	66	V71l	97	79	1062	89	89	106	106	V74l	V169l	228	Y91h	x
12	h	M203	F227	L78	L83	L111	L91	V1074	L107	L101	L118	M118	I90	V177	M244	L103	A1716
13	V	204	228	79	84	I112l	I92l	1075	108	102	I119l	119	91	L178l	245	104	1717
14	E	206	230	81	x	114	94	1077	x	x	121	121	93	180	x	106	x
15	h	A208	A232	L83	L88	V116	V96	M1079	M112	M106	P123	V123	V95	I182	L249	C108	M1721
16	L	x	237	87	92	x	101	1084	116	111	129	M128h	100	187	253	A112l	x
17	h	V236	I259	L111	M115	L140	I123	I1116	M137	L133	V151	I150	A122	I202	L277	A135	M1746
18	h	M240	L263	L115	V119	L144	V127	M1120	L141	I137	V155	F154	L126	V206	L281	L139	F1749
19	H	x	267	119	123	148	131	x	145	141	159	158	130	x	285	143	T1753p
20	h	F248	V271	V123	L127	I152	I135	F1128	I149	I145	V163	L162	I134	I214	I290	M147	A1678
21	h	I249	V272	L124	V128	M153	A136	V1129	I150	V146	L164	I163	I135	V215	I291	I148	x
22	H	250	Y273r	125	Y129r	154	137	1130	151	147	165	Y164r	136	216	292	149	x
23	R	251	274	126	130	155	x	1131	152	148	166	165	137	x	x	150	x
24	D	252	275	127	131	156	139	1132	153	149	167	166	138	218	294	151	1765
25	l	L253	I276	L128	I132	V157	L140	L1133	L154	L150	I168	L167	V139	L219	I295	V152	L1766
26	K	R256b	277	129	133	158	141	R1136b	155	151	169	168	140	S220p	296	153	1727
27	N	257	280	132	136	161	144	1137	158	154	172	171	143	223	299	156	Q1767p
28	h	L258	L281	L133	F137	V162	I145	C1138	L159	I155	I173	L172	I144	V224	V300	I157	x
29	h	L259	M282	L134	L138	M163	M146	M1139	A160	L156	L174	L173	M145	L225	L301	L158	x
30	h	L260	L283	I135	I139	I164	L147	V1140	V161	L157	I175	I174	I146	V226	M302	L159	x
31	l	V266	I289	I141	I150	L171	I157	V1146	L167	I163	L182	I180	V152	I231	I546	V165	x
32	K	267	290	142	Y151p	R172b	158	1147	168	164	183	Q181p	153	x	547	166	N1772p
33	l	I268	I291	L143	V152	L173	I159	I1148	I169	L165	L184	V182	V154	I233	I548	L167	L1773
34	D	270	293	145	154	175	161	1150	171	167	186	184	156	235	550	169	x
35	F	271	294	146	155	W176r	162	1151	172	168	187	185	157	236	L551h	170	P1776h
36	G	272	295	147	156	177	163	1152	173	169	188	186	158	P237s	552	171	x
37	P	299	319	171	x	200	186	1178	194	192	210	207	185	x	573	191	A1806s
38	E	300	320	172	N188p	201	187	1179	195	193	211	208	186	x	574	192	x
39	D	312	332	185	200	214	199	1191	208	211	x	220	198	x	586	207	x
40	W	314	334	187	x	216	201	1193	210	213	226	222	Y200r	x	588	209	x
41	G	317	337	190	205	219	204	1196	213	216	229	225	203	I259l	A591v	212	x
42	h	C367	L384	M266	Y256	L304	L255	C1245	M291	F267	C270	L272	A254	I259	M686	C261	x
43	L	W368h	385	267	M257h	305	256	W1246h	292	268	271	273	255	x	687	262	x
44	R	375	392	274	x	312	263	1253	299	275	278	280	262	x	694	269	x

* ChaK was aligned directly onto the consensus generated from the other fifteen samples. See Results section.

The amino acid or amino-acid category of the conserved residue is listed under “type”. For each structure the residue identifier corresponding to the conserved point is indicated (listed as x if the point is absent). For category types the amino acid present in each structure is indicated before the identifier. If a sample is missing a conserved amino acid but has a similar residue in the same location then the shared category is listed after the identifier. a, acidic; l, aliphatic; r, aromatic; b, basic; c, charged; h, hydrophobic; p, polar; s, small; v, very small.

### Rio2, ChaK and conserved residue variability

**Figure 4:**
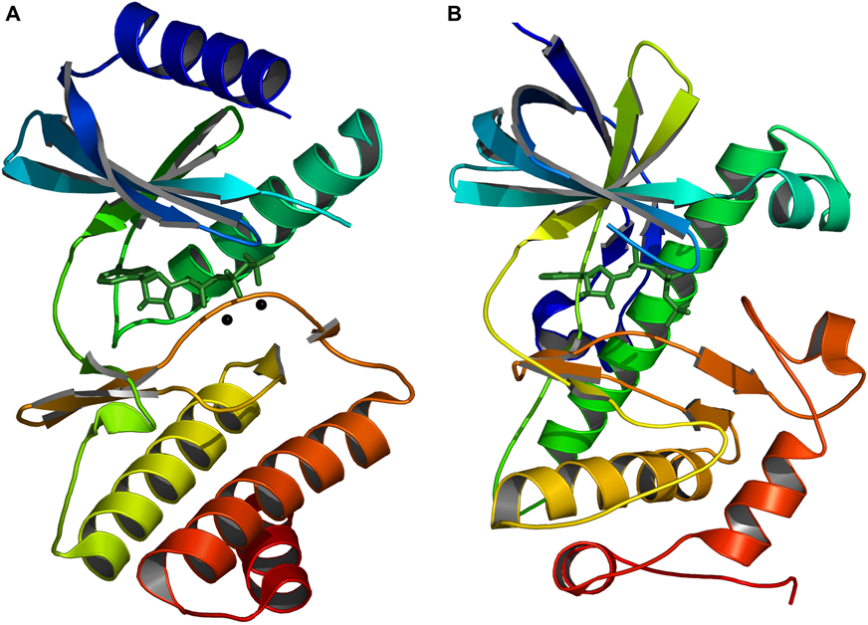
The atypical kinases (A) Rio2 and (B) transient receptor potential channel kinase (ChaK).

Rio2 is the only atypical protein kinase amongst those included in our multiple alignment. No significant sequence similarity can be detected between Rio2 and conventional protein kinases. As such, it is classified with a distinct domain name (see **Table 1**). The structure of the Rio family, as initially determined by [14], shares significant similarity with serine/threonine and tyrosine kinases in the small lobe and in the regions of the large lobe directly adjacent to the active site (**Figure 4A**). Dissimilarity in structure occurs predominantly in the large lobe, in regions involved in substrate specificity, suggesting Rio2 has evolved a novel mechanism of substrate recognition [14]. Our structural alignment algorithm concurs with these findings, showing high similarity in and around the active site (consensus residues 1–36) but not in the large lobe (consensus residues 37–44).

Slightly different results are produced for another atypical protein kinase: channel kinase (ChaK). The structure of this kinase was not included in our initial data set because it lacked a divalent cation in the active site. To examine structural similarity between ChaK and conventional protein kinases, we did align the partially active structure of ChaK (**Figure 4B**) with the consensus structure generated from fully active-conformation kinases (see **Table 2**). Most similarity is found in the region directly around the γ-phosphate of ATP, although there is some elsewhere. The large lobe, like Rio2, is quite distinct. One of the few differences between this kinase and the others is that the fully conserved asparagine 27 is replaced by the very similar carboxamide containing glutamine. The fully conserved aspartic acid 34 is present but does not align, likely due to the absence of a divalent cation.

**Figure 5:**
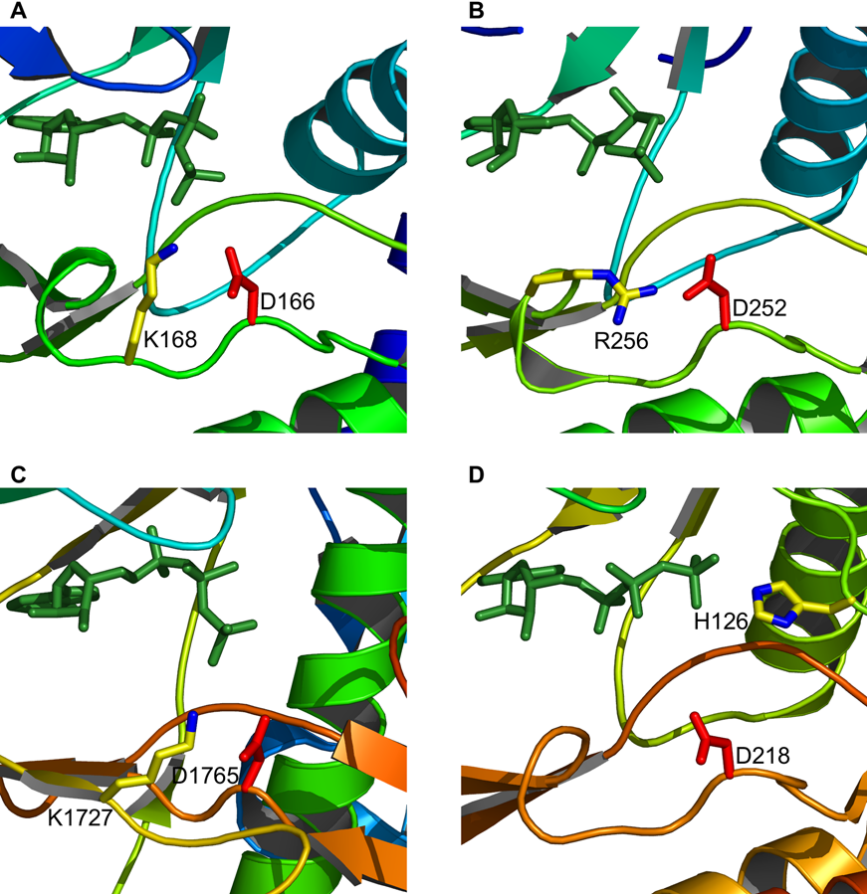
Residue variability in positioning the γ-phosphate of ATP. In each kinase the catalytic aspartic acid is shown in red and the positively-charged residue that interacts with the γ-phosphate of ATP is shown in yellow with nitrogen atoms colored blue. ATP is shown in green sticks. (A) Protein kinase A, (B) activated CDC42 kinase 1, (C) channel kinase, and (D) Rio2 kinase.

We highlight these two kinases for the additional reason that they demonstrate structural variation within the bounds of certain functional constraints. An interesting case of this is lysine/arginine 26. In almost all serine/threonine kinases there is a lysine residue located two positions downstream of the catalytic aspartic acid (consensus residue 24). This lysine aids in orientating the γ-phosphate of ATP and it is thought may also act to neutralize the negative charge on this phosphate during catalysis. The position and orientation of these two residues with respect to ATP is shown in protein kinase A (PKA) in **Figure 5A**. Tyrosine kinases, like activated CDC42 kinase (ACK1), do not have this lysine but instead have an arginine four positions downstream from the catalytic residue (**Figure 5B**). This residue is orientated perpendicular to the lysine but occupies the same location, and since both are positively charged basic residues, both can fulfill the same function. ChaK, a serine/threonine kinase like PKA, utilizes a lysine for this function, which again occupies the same geometric position (**Figure 5C**). However, this residue is not located two or four positions downstream, but thirty-eight positions upstream on a β-strand running adjacent to the catalytic loop. This is a good demonstration of the value behind a sequence-order independent alignment algorithm that ignores fold and residue connectivity. This lysine is located on part of a novel fold not found in any of the other kinases examined. Rio2 presents a fourth variation. Our alignment did not reveal Rio2 as having a basic residue at consensus point 26, as it did for all of the other kinases. Further examination led us to the conclusion that the function of this residue is conserved in Rio2 but the location of the residue accomplishing it is not (see also [14]). This is known as functional residue hopping [15], [16]. The function of consensus residue 26 is performed by a histidine located in the small lobe (**Figure 5D**). This histidine is largely unique to the *Archaeoglobus fulgidus* Rio2 ortholog, from which the structure was derived – in most other species it is substituted by an arginine. As *A. fulgidus* is a hyperthermophile, the preference for histidine may be due to the extreme temperature environments in which it is found.

**Figure 6:**
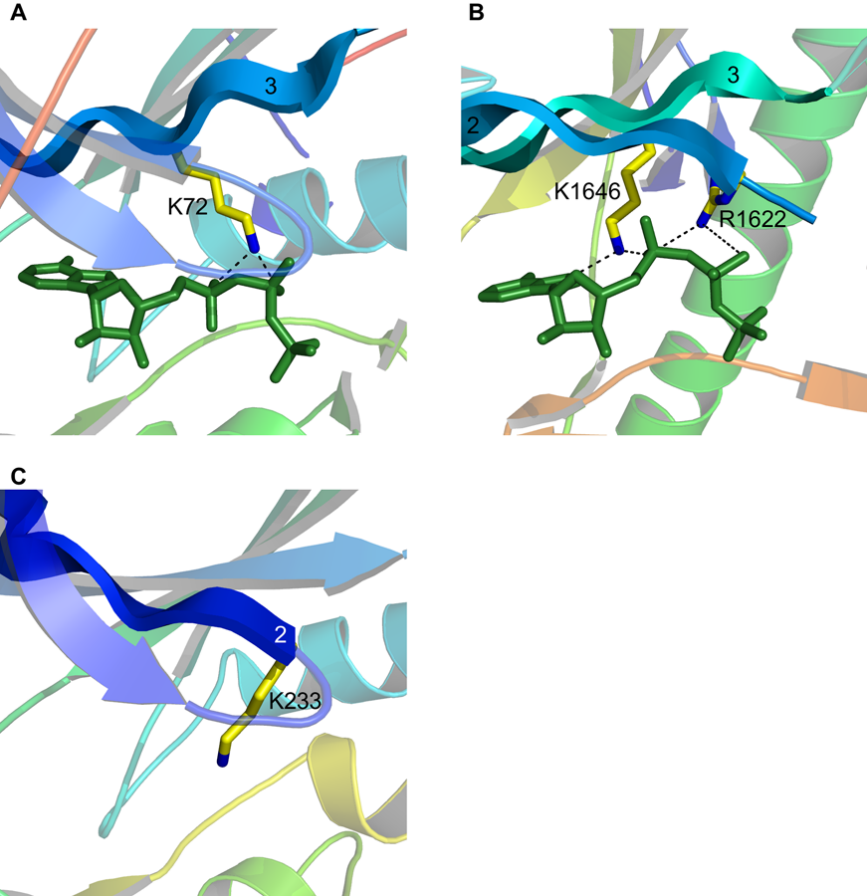
Variations in the catalytic lysine. (A) In almost all protein kinases (such as protein kinase A shown) a lysine residue originating from β-strand 3 interacts with the α- and β-phosphates of ATP. This lysine is required for catalytic activity and has been termed the catalytic lysine. (B) In channel kinase (ChaK), the homologous lysine interacts with the α-phosphate and the adenine ring of ATP. An unique arginine residue located on β-strand 2 instead interacts with the α- and β-phosphates. (C) In protein kinase with no lysine 1 (WNK1, PDB code: 1T4H [22]), the catalytic lysine is present but originates from β-strand 2, much like the arginine from ChaK. ATP is shown in green sticks. Potential hydrogen bonds between the positively charged residues and ATP are shown as dashed lines, and nitrogen atoms are colored blue. The WNK structure has no ATP or ATP analog.

The only other variability we have found in conserved residues is for the so-called catalytic lysine (lysine 8). The function of this residue is somewhat debated [17], [18], [19], but at the very least it appears to position the phosphates of ATP and is absolutely crucial for catalysis. This residue is normally found on β-strand 3 and interacts with the α- and β-phosphates of ATP (**Figure 6A**). In ChaK there is a homologous lysine present but it interacts with the α-phosphate and the adenine ring of ATP (**Figure 6B**). Depending upon the alignment parameters used, this lysine can align structurally with that found in other kinases, although for the parameters we have chosen it does not. Instead there is an arginine residue that aligns and this very similar residue interacts with both the α- and β-phosphates of ATP (**Figure 6B**), just as the catalytic lysine normally does. Although ChaK lacks a divalent cation, this is unlikely to affect the positions of these residues. The arginine in question, R1622, is fully conserved in the alpha kinase family [20]. It is not known which residue in ChaK is functionally homologous to the catalytic lysine in typical protein kinases. Likely both share the function, and this represents a distinct feature of the alpha-kinase family. One other kinase is known to have a novelty in this area. This is the protein kinase with no lysine (WNK) kinase, named for the apparent absence of the catalytic lysine on β-strand 3. It was predicted by [21] that a lysine on β-strand 2 could be structurally equivalent (**Figure 6C**), and the absolute requirement for a lysine at this position was confirmed by this group. A subsequent crystal structure appears to confirm these predictions [22]. It is interesting that this lysine originates from the same position as the arginine in ChaK, showing that variation in conserved residues is possible and highlighting how proteins can incorporate novel features within certain functional constraints.

### Substrate specific variation

The conservation we are detecting is found first and foremost in and around the active site, but is present elsewhere in the protein kinase domain. The single exception is in the cleft formed by the catalytic loop, the P+1 loop, helix D and the residues from the end of helix F through to the beginning of helix H. This is known as the substrate-binding cleft, and residues in this region have been shown crucial to substrate binding and regulatory protein-protein interactions [23], [24], [25], [26], [27], [28], [29]. The absence of conservation in this cleft is entirely expected. Protein kinases are substrate specific and that specificity is at least in part conferred through residue variability within this groove.

**Figure 7:**
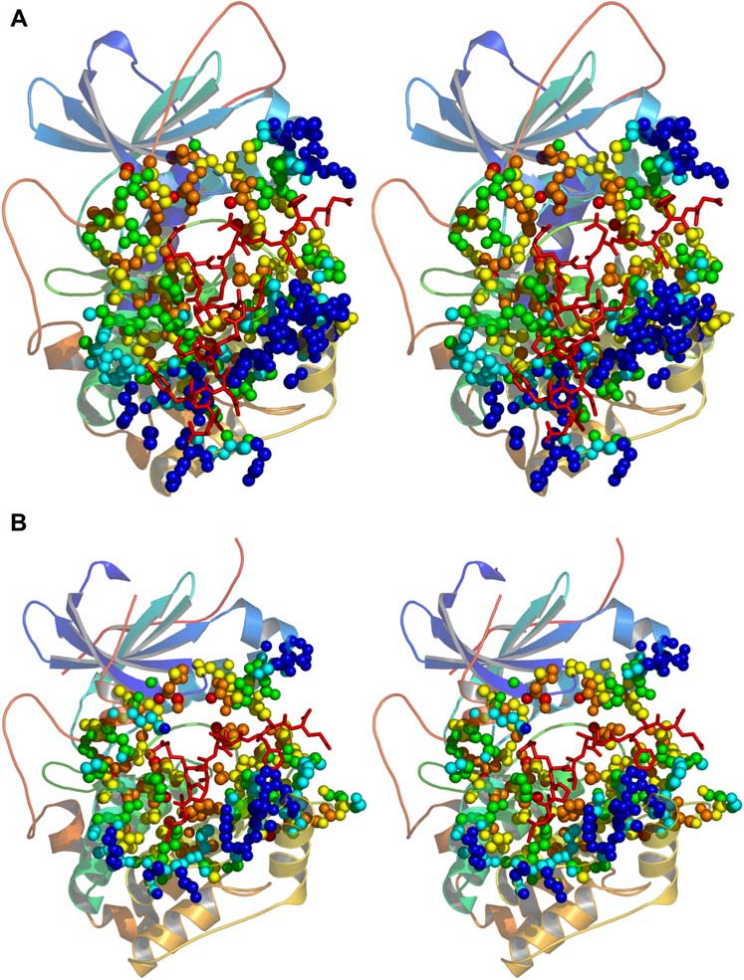
Substrate-specific variability. Cross-eyed stereo views of (A) protein kinase A and (B) Akt2. Bound substrate peptides are shown in red sticks. Atoms belonging to non-conserved residues within 10 Å of the substrate peptide are shown as colored spheres. Red: atoms within 2 Å of a conserved residue; orange: atoms between 2 and 4 Å of a conserved residue; yellow: atoms between 4 and 6 Å of a conserved residue; green: atoms between 6 and 8 Å of a conserved residue; cyan: atoms between 8 and 10 Å of a conserved residue; and blue: atoms more than 10 Å from a conserved residue.

The crystal structures of PKA and Akt we have used in our analysis contain substrate peptides bound in this region. In **Figure 7** we show PKA and Akt with bound substrate peptides and display variability in the context of proximity to the substrate and distance from conserved residues. In both PKA and Akt, there are a number of atoms in the substrate-binding cleft adjacent to or near the substrate peptide that are distant from conserved residues. We highlight this for the purpose of discussing inhibitor design. Successful inhibitor design requires a region where small molecules or peptides can be bound with high specificity. The absence of specificity comes from drug targets hitting regions of residue conservation. The substrate-binding cleft obviously has a binding capacity and, as shown, this region is highly variable. Designing inhibitors that target the atoms in this region, mimicking those residues present in substrates or regulatory proteins, would be a fruitful approach.

### Structure prediction

The conservation we are finding can be used for purposes other than understanding protein function, evolution and guiding drug design. It can also be used to predict the structure of protein kinases (and to other proteins if applied). The consensus structure shown in **Figure 3** represents the typical position of specific and conserved amino acids or amino-acid categories. If this is a distribution that active protein kinases tend to adopt, then predicted structures of active-conformation protein kinases should be made to meet these criteria. At the most basic level, models can be generated through any method and then screened against a consensus structure to discriminate between good and bad models, or, alternatively, residues known to be equivalent from a sequence alignment can be forced to meet the constraints seen in the consensus structure and the rest of the protein can be modeled within this framework. We have explored these possibilities with the structure prediction method Rosetta [30] and attempted to model two kinases, the typical 3-phosphoinositide dependent protein kinase-1 and the atypical Rio2 kinase.

**Figure 8:**
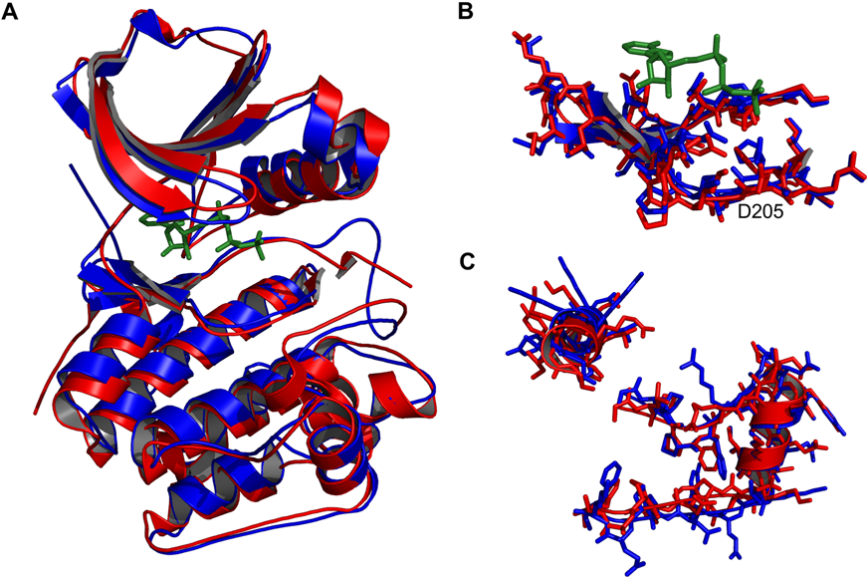
Modeling a typical kinase. PDK1 was modeled using the consensus guided approach outlined in the Results and Methods sections. (A) The best model is in blue and the actual structure in red (PDB code: 1H1W). Structures are shown aligned based on C_α_ RMSD. (B) Residues 201–225, comprising the floor of the active site, are highlighted as aligned on side chains. (C) Residues from the non-conserved regions of the substrate binding groove (residues 166–175 and 278–305) are shown as aligned on side chains. ATP from the true structure of PDK1 is shown in green sticks.

We used fourteen active-conformation kinases (omitting Rio2) and generated a 52 residue consensus structure (consensus residues are listed in **Supplementary Table S1**). A sequence alignment of these fourteen kinases and PDK1 was then generated using ClustalW [31]. Residues from PDK1 apparently equivalent with those of the consensus were selected based on this sequence alignment (see **Supplementary Table S1**). These residues were then constrained geometrically in accordance with the consensus while PDK1 was modeled with Rosetta as described in the Methods section. When compared against the partially active-conformation structure of PDK1 (PDB code: 1H1W, [32]), the top ranked prediction had 198 of 285 side chains positioned within 2 Å of their actual location, a C_α_ RMSD of 1.3 Å and an all-atom RMSD of 1.6 Å (**Figure 8A**). The floor of the active site, where most conservation occurs, is highly accurate, with 24 of 25 side chains positioned with 2 Å, C_α_ RMSD of 0.5 Å and an all-atom RMSD of 0.7 Å (**Figure 8B**). In non-conserved regions, such as the substrate-binding groove, good modeling is dependent upon the ability of the prediction method applied. A well-proven method like Rosetta is, therefore, a good complement. 24 of 38 residues in the substrate-binding groove are within 2 Å of their actual position, a C_α_ RMSD of 0.9 Å and an all-atom RMSD of 1.4 Å (**Figure 8C**). Accuracy in this region may be due in part to the constraints applied elsewhere, which would reduce the potential conformational space to search.

**Figure 9:**
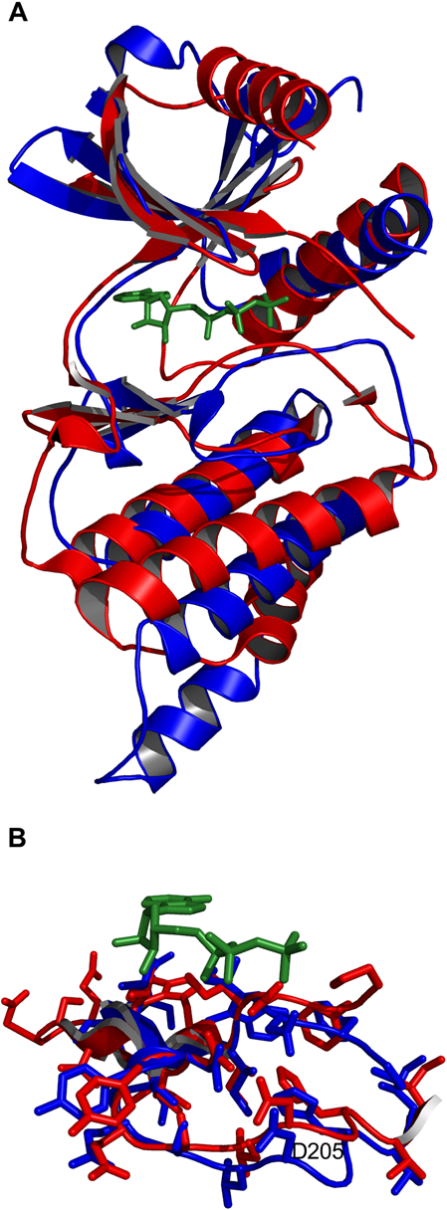
Modeling an atypical kinase, Rio2. (A) The best model is in blue and the actual structure in red (PDB code: 1ZAO). Structures are shown aligned based on C_α_ RMSD. Residues 128–142 from the model are hidden because they are disordered in the actual structure. (B) Residues 214–237 are highlighted as aligned on side-chains. ATP is shown in green sticks.

Rio2 kinase was modeled initially without constraints as these cannot be derived from a sequence alignment. 80,000 models were generated and the lowest 5% (in full atom energy) were screened against the consensus structure. The top ten were then scrutinized for potential constraints. All ten of the top models unambiguously agreed on the likely equivalent residues for the fully conserved lysine 7 (K120 in Rio2), aspartic acid 24 (D218), asparagine 27 (N223) and aspartic acid 34 (D235). In none of the models could a residue equivalent to the fully conserved glutamic acid 8 be found (it should be E154). We then proceeded with a second modeling phase using constraints from the consensus for K120, D218, N223 and D235, and allowing two candidates for the conserved glutamic acid: E134 or E154. Although models generated with E134 as the conserved glutamic acid scored equally well when compared against the consensus structure, they would likely be deemed implausible by visual inspection as helix C was distorted upwards away from the active site instead of lining the back of the ATP binding pocket as it does in other kinases. The best model for E154 as determined against the consensus structure is shown in **Figure 9A** alongside the actual active-conformation structure (PDB code: 1ZAO). Fifty four of 180 side-chains are positioned within 2 Å of their true location. C_α_ and all-atom RMSD are 6.1 Å and 7.1 Å respectively. However, these values do not accurately reflect the quality of the model. The N-terminus of protein kinases is a non-conserved region and the C-terminal lobe of Rio2 differs greatly from that of other kinases. It should not be expected that a structural-based consensus approach would be able to distinguish a correct model from an incorrect model in these regions. Looking solely at the conserved kinase regions, comprising residues 93–242 of Rio2, the C_α_ and all-atom RMSD drop to 3.0 Å and 3.7 Å respectively. And all of the correctly positioned residues are found in this region. The floor of the active site has 16 of 24 side chains positioned with 2 Å, C_α_ RMSD of 1.0 Å and an all-atom RMSD of 1.7 Å (**Figure 9B**). A final note: although residues located in the non-conserved large lobe helices are not correctly positioned, this region is topologically correct.

## Discussion

The kinase domain can be conceptualized as two functional modules. A highly conserved ATP-binding and catalytic module, located between the small and large lobes, found in all typical and atypical protein kinase structures. Little variation is found here, with only a few kinases, such as Rio2, ChaK and WNK, containing structural (but not functional) novelties. The second module is involved in substrate binding and evidence suggests is localized primarily to the large lobe, in the region named the substrate/peptide binding groove (named from kinase-peptide co-crystal structures). Very little is known about this region of kinases as detailed knowledge would require many kinase structures co-crystallized with full-length substrates, and as yet no single such structure exists. Conservation of the groove fold suggests a common substrate-binding mechanism, while the absence of residue conservation in this region across the kinase family suggests the means by which substrate specificity is determined. The part of this groove located near the active site (between the catalytic and P+1 loops) does contain conserved residues and is entirely consistent with the broad peptide-substrate specificity of kinases. But peptides are not the same as full-length proteins, and studies on the mitogen-activated protein kinase p38 have shown that proteins can be phosphorylated at a hundred to thousand fold greater rate than peptides [33]. More than just the residues immediately attached to and surrounding the target serine, threonine or tyrosine are involved in the kinase-substrate interaction, and the general mechanism of interaction needs to be determined.

Although the substrate/peptide binding groove may be the key feature for understanding substrate specificity in most kinases, it is and need not be in all. The substrate-binding module can vary in three ways: typical kinases that preserve fold and merely vary the residues in the substrate-binding groove, typical kinases than rely on other regions/regulatory proteins in addition to the substrate-binding groove, and atypical kinases that vary the binding mechanism all together. It should be added that even in very typical kinases substrate interactions are not localized purely to the substrate-binding groove (see [34]). Much work needs to be done on this area of protein kinases, and current knowledge of substrate-binding mechanisms is rudimentary.

A thorough understanding of substrate interactions will help in discovering new proteins targeted by kinases. Insights into binding can guide computational docking approaches that test the fit of different structures onto kinases as a new means of substrate finding. More experimentally determined structures or advances in structure prediction would be a necessary requirement for this to have broad applicability. This may be a ways off yet, but a closer goal may be in applying structural binding information to the design of specific kinase inhibitors. Currently, inhibitors are primarily designed to target the ATP binding pocket, the region of greatest conservation amongst kinases. The ATP binding pocket does contain unique features between certain subsets of the kinome (the gatekeeper residue is an example [35], [36]), and these novelties can be exploited by drugs. However, this type of design approach will always be plagued by problems of specificity on the simple foundation that this region has evolved to bind a single thing: ATP. On the other hand, the substrate-binding groove has evolved to bind kinase-specific substrates and our analysis demonstrates that this is accompanied by large variability in the substrate-binding groove. Mimicking these substrates with peptides or small molecules that compete with substrates has proven effective in several cases (reviewed in [37]), and a peptide inhibitor that mimics a substrate interaction domain has been shown useful *in vivo* at reducing tumor mass [38].

Of great benefit in this regard would be data on kinase substrates. If wide-ranging data on kinase substrate pools were known, inhibitors could be designed to mimic exclusive targets. Or if there was a desire to inhibit multiple kinases at one time, a small molecule that mimics a common substrate could be made. Ultimately the viability of such a strategy would again require a greater output of crystal and NMR structures, advances in structure prediction and protein docking, but these are popular areas of research and should not prove a hindrance. A dendrogram classification of kinases based upon substrates would be of tremendous use for inhibitor design, cell biology and evolutionary studies. Such an aim would require systematic methods of substrate finding, which are beginning to become available [39], [6].

Protein structures are the key to what we have presented and are discussing. Unless there are significant advances in the rate and ease with which proteins can be cloned, expressed, purified and structures subsequently determined, reliable computational approaches at structure determination will be needed. We have found that structural conservation gives insight not only into protein function but also can be used with success for structure prediction. We have shown how knowledge of residue conservation can help in generating or selecting appropriate protein kinase models. The atypical Rio2 kinase, which possesses no significant sequence similarity to other protein kinases, would be a difficult target for standard approaches at homology modeling. As few as five geometric residue constraints derived from a consensus structure and further screening can select a highly accurate model. For a typical kinase, such as PDK1, where many constraints can be used (50 in our case), it was a simple matter to generate a highly accurate model. Residue conservation and other types of structural similarity quantified over protein families can provide the basis for model selection and act as guides that reduce the target search space for the computationally demanding task of structure prediction.

Understanding the individual role of every protein kinase in the genome is a daunting task, but necessary because of the important signaling role played by this family of enzymes and the potential for disregulation and subsequent disease. Ultimately, cellular studies that dissect the role of kinases *in vivo* and the treatment of disease will require a library of specific and multi-target inhibitors. Such an aim will require a strong union between computational, molecular and structural approaches, but this goal may be close at hand. Although empirically determined structures of all kinases are a long way off, quality models can be built. Kinome wide data on substrate pools will require a great deal of work, but the techniques are now becoming available. The mechanism(s) of substrate binding will need to be elucidated in detail. This may be the greatest challenge, and towards which maximum focus should be directed, as not a single kinase-substrate structure exists. But, if such can be achieved in a representative sample of cases, kinase-substrate specific docking algorithms may be able to do the rest. The attainment of these aims would allow for the quick and reliable design of specific inhibitors, thereby allowing the precise function(s) of kinases to be determined and creating the means by which signaling networks can be precisely manipulated.

## Materials and Methods

### Structural alignment overview

The results presented in this paper were generated through the use of a multiple structure alignment algorithm we have developed. The motivation was to have a method for superimposing protein structures independent of residue connectivity, thereby facilitating comparisons between proteins that have distinct folds and maximize side-chain as opposed to main-chain overlap. The field of shape analysis, specifically the Procrustes methods, provided the foundation for accomplishing this task. What follows is an overview of Procrustes and a description of its modification and application to protein structure comparison as we have implemented it.

### Procrustes

Procrustes is a method for comparing matrices and thereby any group of objects that can be represented in such a way [40], [41]. It is part of the broader field of shape analysis which has its motivation in describing and understanding variation between objects through multivariate analysis. Procrustes is often applied in biological and anthropological studies of morphology, for example in studying the relationship between turtle skull shape and life style across species [42]. It has been used in agriculture, genetics, geography, geology and psychology to name a few fields. Although multivariate analysis is not of interest, the Procrustes method is valuable for its ability to efficiently superimpose objects. Procrustes superimposition initiates from a set of points, each of which has a representative in every object under consideration. These points, known as landmarks, must have some level of correspondence and be located within a geometric space. By manipulating scale, location and orientation, Procrustes aims to produce an optimal superimposition as defined by some criteria that acts to minimize distance between corresponding points. The term partial Procrustes specifically refers to the subset of methods that maintain scale, i.e. those approaches that only perform rigid body transformations [43].

### Application and algorithm

An application for Procrustes in protein structure alignment is evident, where landmarks could be viewed as equivalent residues in a space of three dimensions. The term equivalent accounts for functional equivalence and/or homology, although emphasis should be placed on the former. Specifically it is the partial Procrustes method that is applicable since initial aspects of scale should be maintained in the resulting alignment.

Standard implementations of Procrustes require a set of user-specified landmarks with representatives in every input object. These two conditions are inadequate for multiple structure alignments. The primary motivation behind a structural alignment should be to find conserved residues, and a good algorithm must give leeway for less than total conservation. The algorithm we have developed allows for both. It proceeds in two phases. The first is a series of pair-wise comparisons to find residues conserved across all samples. Residue conservation means in terms of both location and amino-acid type (identity/similarity). The second phase begins with an initial multiple alignment using the landmarks found in the first phase. From the multiple alignment the set of landmarks is then extended to include any additional fully conserved residues not found in the first phase, and residues with less than total conservation. The multiple alignment and extension steps are repeated until a final non-extendable set is obtained or upon completion of a predefined number of iterations.

In this approach, each of *n* structures is represented by a *l_i_*×3 coordinate matrix, where *l_i_* equals the number of residues in structure *i*. The mean of the non-hydrogen side chain atoms is used as the coordinate for each residue, with the exception of glycine residues for which the alpha carbon (C_α_) is used. Most structure alignment algorithms use the C_α_ for all residues. This is inadequate. Residue function should be viewed largely in terms of side-chain characteristics: what atoms make up the side chain and where they are positioned. It is the side chain that is principally used to interact with ligands, substrates and other residues [44], making knowledge of its position the crucial feature. Superimposed C_α_'s of similar residues may have side chains positioned in different locations and are therefore unlikely to have the same function. For these reasons focus is placed on side-chain positioning.

One object (structure) can be superimposed onto a second in three dimensions if at least three equivalent residues are known. It is possible these residues may or could be known beforehand, although this is not ideal. An alternative involves a search and score approach: choose three residues from each sample that have corresponding amino-acid labels and share some geometric feature, then score the alignment produced from superimposing these residue triplets. The highest scoring alignment produced from a series of triplet superimpositions can then be used to produce a set of residues conserved across a pair of samples (we term this a pair-wise consensus set). This approach has been employed in a hash table. For all samples every triplet of residues is stored based on the amino-acid labels of its members and the inter-residue distances are indexed. Each entry of the table can be thought of as a triangle with vertices labeled according to amino acid. Between pairs of samples highly congruent triangles with matching vertices are used as candidate triplets likely to produce high scoring superimpositions. Such criteria reduce the time spent on triplets least likely to be part of the final pair-wise consensus set. We have used congruency criteria of 1–10% as indicated below, i.e. corresponding vertices must have magnitudes within 1–10% of each other.

The method of superimposition is taken from Procrustes. Candidate triplets from each sample *i* are stored in an 3×3 coordinate matrix *X_i_*. These matrices are first Helmertized (centered), removing translational differences:
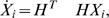
(1)where 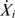 is the centered matrix and *H* the (*m*−1)×*m* Helmert sub-matrix whose *j* ^th^ row is equal to (*h_j_*,…, *h_j_*, −*jh_j_*,0,…,0) for *h_j_* = −[*j*(*j*+1)]^−1/2^. Other approaches for removing translation are available. The optimal rotation of 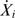 onto 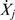 is found by minimizing
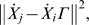
(2)where Γ is a 3×3 rotation matrix. It can be shown that if *VΔU^T^* is the singular value decomposition of 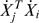, the optimal rotation Γ = *UV^T^* (provisions must be made to ensure Γ does not also encompass a reflection).

The translation resulting from centering triplets is then applied to each structure and the appropriate sample is rotated by Γ. This superimposes the equivalent triplet residues and pulls the rest of each structure along. The result is that the initial pair-wise consensus set a triplet comprises may be extended from the structure-wide superimposition. Residues from the superimposed structures are considered to be equivalent (i.e. in the pair-wise consensus set) if they are identical or similar amino acids within two angstroms of one another. Similar means sharing a category, with those allowed including acidic (D, E, Z), aliphatic (A, G, I, L, V), aromatic (F, H, W, Y), basic (H, K, R), charged (D, E, H, K, R, Z), hydrophobic (A, C, F, G, I, L, M, P, V, W, Y), polar (C, D, E, H, K, N, Q, R, S, T, Y, Z), small (A, C, D, G, N, P, S, T, V) and very small (A, G, S), where Z refers to a phosphorylated serine, threonine or tyrosine.

This procedure is performed on all triplets that match the congruency criteria and all alignments are scored based on the frequency with which the amino acids of any equivalent residues occur. The frequency of occurrence for each amino acid was obtained from the protein knowledge-base release statistics of Swiss-Prot (Release 49.5, 18/04/06). Tryptophan is the lowest occurring amino acid and superimposed tryptophans are therefore given a score of one. All other scores are relative to that of tryptophan: score(amino acid x) = occurrence(W/x). The occurrence of each category is equal to the sum of the occurrences of its members. For *m* equivalent residues, the alignment score is
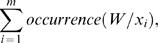
(3)where *x_i_* is the amino acid or amino-acid category of the *i* ^th^ equivalent residue.

The top 100 scoring triplets are subjected to a second phase of testing to find maximum similarity between two structures. For these triplets the initial pair-wise consensus set is used for a second superimposition. In this case the *X_i_* coordinate matrices take on dimensions *m*×3, for *m* equivalent residues in the pair-wise consensus set. The superimposition and extension steps are repeated until a final non-extendable set is obtained.

Computational time and memory are minimized in three ways. First, as has been mentioned, congruency between triangles is used to reduce the number of triplets tested. Second, the vertices of these triplets are only indexed by amino acid and not by category as well. If vertices could be indexed by amino-acid category, such as hydrophobic, the number of candidate triplets to search scales tremendously, as does demands upon memory if a hash table is employed. Third, we restrict triplets to a core of residues. For a structure of 300 residues there are nearly 27 million candidate triplets. Storing all in a hash table is too demanding, and is not necessary either. The best candidate triplets will be clustered in a core, which may be comprised of residues around an active site, ligand binding domain or phosphorylation site. Only searching this core will provide good candidate triplets and minimize time. By default a geometric core of fifty residues is used.

From the input structures a consensus set *S_n_* is sought for of residues fully conserved across all *n* samples (a full consensus set). This can be done quickly through a series of pair-wise comparisons. First a consensus set *S_2_* of conserved residues is sought for between samples 1 and 2 using the superimposition procedure outlined above. A second set *S_3_* of conserved residues found in *S_2_* and sample 3 is then sought for using the same procedure of selecting triplets from the consensus set and the sample. This process is repeated until a final full consensus set *S_n_* is produced. From experience the procedure for deriving *S_n_* is generally sample order independent, except in some cases where an extreme outlier is present. This is a structure with few of the conserved residues found across the other *n*−1 samples. In this case the outlier may align well with a set of residues conserved across the first *x* samples but not across the final *n*−*x*−1. By aligning well with a subset of *S_x_* not in *S_n_*, the outlier skews the derivation process away from the true set *S_n_*. This can be accounted for by leaving such outliers until the end, by which time the consensus set has been reduced to a true group of highly conserved residues.

The multiple alignment procedure is a modification of the pair-wise superimposition method outlined above. The full consensus set *S_n_* found through the pair-wise superimpositions is comprised of a set of *m* landmarks that can be used to simultaneously align the group of *n* structures. First, the conserved residues from each sample *i* are stored in an *m*×3 matrix *X_i_* and Helmertized. The optimal rotation matrix for each structure Γ*_i_* is calculated by an iterative process that seeks to minimize the following value:
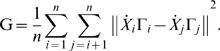
(4)The rotation Γ*_i_* is calculated from the singular value decomposition of 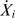 with the mean of the rest, 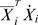, for
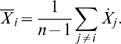
(5)However, with each successive 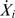 rotated, the previous 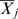 for *j*<*i* will change and the previously calculated rotation of 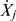 will be incorrect. To account for this, after every sample has been rotated *G* is calculated and the process of rotation is repeated until the change in *G* falls below some arbitrary small threshold (i.e. convergence occurs). For *p* steps until convergence, the optimal rotation of each structure is equal to the product of the successive Γ*_i_* rotations.

This procedure superimposes the conserved residues found in the set *S_n_*. The next goal is to extend this set. Multiple alignment is more accurate than a series of pair-wise alignments and this allows for *S_n_* to be extended to include additional fully conserved residues. At the same time residues with less than full conservation can be incorporated. We have defined conserved residues as identical or similar amino acids from different structures within two angstroms of some point, termed a consensus point. Consensus points are considered redundant if they contain more than 50% of the same residues. The point closer to the relevant landmarks is retained. Partially conserved residues must only be present in a threshold of samples and the less than full conservation of an amino acid takes precedence over the conservation of a category. Leeway in the conservation of residues allows for evolutionary freedom, but is also required for practical reasons such as x-ray resolution, model construction, side-chain movement and non-significant sample-dependent positionings. As the superimposition procedure requires all landmarks in all samples, any samples with missing landmarks are temporally given the consensus point as a place filler. If new residues can be added following an alignment then the expanded set *S_n_* is used to re-align the structures. The extension and alignment process is repeated until a final consensus set is converged upon or until the completion of a predetermined number of iterations.

### Aligning active-conformation kinases

Active-conformation kinases were aligned using the above procedure with a congruency criteria of 1% and a distance constraint for conservation of two angstroms. The partially active-conformation structure of the transient receptor potential channel kinase was aligned directly onto the consensus using congruency criteria of 10% and a distance constraint of 2.25 angstroms.

### Availability and Performance

The program is written in C and Fortran. Full source code is freely available under the GNU General Public License by contacting the authors. PDB formatted files are required for input. For multiple alignments output consists of PDB files modified according to the alignment, a consensus structure in PDB format, a csv file indicating the conserved and aligned residues, and a script for viewing the alignment and consensus in the open-source program PyMOL (http://www.pymol.org). For a pair-wise alignment against a consensus structure, output is as above except no consensus structure is produced. The alignment and consensus structure of the fifteen kinases presented in the Results section were generated in ∼twenty seconds using a single processing core of an AMD Athlon 64 X2 4200 processor with 1GB of RAM. A single pair-wise alignment takes ∼2–8 seconds depending upon the degree of similarity between input samples. Comparisons between a sample and a consensus structure are very rapid (<1 second due to the reduced set of residues in a consensus set).

### Structure prediction

Constraints from fourteen active-conformation kinases were generated for both the side-chain and the α-carbon. Constraints were derived from the final structural alignment. Side-chain constraints were applied to the atom closest to the median of all side-chain atoms. Structure models were created following the Rosetta homology modeling protocol described in Das *et al*. [30]. During full-atom refinement of a model, a penalty score is applied when the atom-atom distances in the model exceed the upper or lower limit of the corresponding distance constraints. If a distance exceeds the upper or lower constraint limit by *d* Angstrom, then the penalty score *Ec* is d*d when *d*<0.5 and *d*−0.25 when *d*≥0.5. For PDK1, the lowest scoring model (in full atom energy) as output by Rosetta was selected as the best model. For Rio2, the lowest 5% of models were used for screening against the consensus structure as described in the Results section. Models were ranked based on their score from Equation.

## Supporting Information

Table S1 (0.23 MB DOC)
