# Supplementary material for: Conservation, Variability and the Modeling of Active Protein Kinases
Source: PLoS One. 2007 Oct 3;2(10):e982. doi: 10.1371/journal.pone.0000982 (PMC1989141; doi:10.1371/journal.pone.0000982)
Supplement: Table S1 — (0.23 MB DOC) [file pone.0000982.s001.doc]

**Supplementary Table 1**: Conserved residues for constraint modeling.

|  | type | ACK1 | Akt2 | CDK2 | CK1 | CK2 | DAPK | IRK | p38γ | PhK | Pim-1 | PKA | PknB | Sky1P | TAO2 | PDK1^*^ | Rio2^*^ |
| --- | --- | --- | --- | --- | --- | --- | --- | --- | --- | --- | --- | --- | --- | --- | --- | --- | --- |
| 1 | h | L132 | L158 | I10 | I18 | V45 | L19 | L1002 | V33 | L25 | L44 | L49 | L17 | L164 | I34 | L88 (CG) | x |
| 2 | G | 133 | 159 | 11 | 19 | 46 | 20 | 1003 | x | 26 | 45 | 50 | 18 | 165 | 35 | 89 (CA) | x |
| 3 | G | x | 161 | x | 21 | 48 | 22 | 1005 | x | 28 | x | 52 | 20 | 167 | 37 | 91 (CA) | x |
| 4 | V | 140 | 166 | 18 | I26l | 53 | 27 | 1010 | 41 | 33 | 52 | 57 | 25 | 172 | 42 | 96 (CB) | x |
| 5 | A | 156 | 179 | 31 | 39 | I66l | 40 | 1028 | 54 | 46 | 65 | 70 | 38 | 185 | 55 | 109 (CB) | x |
| 6 | h | V157 | M180 | L32 | I40 | I67 | A41 | V1029 | I55 | V47 | I66 | M71 | V39 | M186 | I56 | I110 (CG1) | x |
| 7 | K | 158 | 181 | 33 | 41 | 68 | 42 | 1030 | 56 | 48 | 67 | 72 | 40 | 187 | 57 | 111 (CD) | x |
| 8 | E | 177 | 200 | 51 | 55 | 81 | 64 | 1047 | 74 | 73 | 89 | 91 | 59 | 202 | 76 | 130 (CD) | 120 (CD) |
| 9 | L | M181h | 204 | 55 | Y59h | 85 | 68 | M1051h | 78 | 77 | 93 | 95 | A63l | 206 | 80 | M134h (CG) | 134/154 (CD) |
| 10 | L | 192 | 215 | 66 | V71l | 97 | 79 | 1062 | 89 | 89 | 106 | 106 | V74l | 228 | Y91h | 145 (CG) | x |
| 11 | h | M203 | F227 | L78 | L83 | L111 | L91 | V1074 | L107 | L101 | L118 | M118 | I90 | M244 | L103 | F157 (CG) | x |
| 12 | V | 204 | 228 | 79 | 84 | I112l | I92l | 1075 | 108 | 102 | I119l | 119 | 91 | 245 | 104 | x | x |
| 13 | E | 206 | 230 | 81 | x | 114 | 94 | 1077 | x | x | 121 | 121 | 93 | 247 | 106 | x | x |
| 14 | h | A208 | A232 | L83 | L88 | V116 | V96 | M1079 | M112 | M106 | P123 | V123 | V95 | L249 | C108 | A162 (CB) | x |
| 15 | L | x | 237 | 87 | 92 | x | 101 | 1084 | 116 | 111 | 129 | M128h | 100 | 253 | A112l | 167 (CG) | x |
| 16 | h | V236 | I259 | L111 | M115 | L140 | I123 | I1116 | M137 | L133 | V151 | I150 | A122 | L277 | A135 | I189 (CG1) | x |
| 17 | h | A237 | V260 | L112 | L116 | L141 | L124 | A1117 | L138 | L134 | L152 | V151 | C123 | L278 | L136 | V190 (CB) | x |
| 18 | h | M240 | L263 | L115 | V119 | L144 | V127 | M1120 | L141 | I137 | V155 | F154 | L126 | L281 | L139 | L193 (CG) | x |
| 19 | H | x | 267 | 119 | 123 | 148 | 131 | x | 145 | 141 | 159 | 158 | 130 | 285 | 143 | 197 (CG) | x |
| 20 | h | F248 | V271 | V123 | L127 | I152 | I135 | F1128 | I149 | I145 | V163 | L162 | I134 | I290 | M147 | I201 (CG1) | x |
| 21 | h | I249 | V272 | L124 | V128 | M153 | A136 | V1129 | I150 | V146 | L164 | I163 | I135 | I291 | I148 | I202 (CG1) | x |
| 22 | H | 250 | Y273r | 125 | Y129r | 154 | 137 | 1130 | 151 | 147 | 165 | Y164r | 136 | 292 | 149 | 203 (CG) | x |
| 23 | R | 251 | 274 | 126 | 130 | 155 | x | 1131 | 152 | 148 | 166 | 165 | 137 | x | 150 | 204 (NE) | x |
| 24 | D | 252 | 275 | 127 | 131 | 156 | 139 | 1132 | 153 | 149 | 167 | 166 | 138 | 294 | 151 | 205 (CG) | 218 (CG) |
| 25 | l | L253 | I276 | L128 | I132 | V157 | L140 | L1133 | L154 | L150 | I168 | L167 | V139 | I295 | V152 | L206 (CG) | x |
| 26 | K | R256b | 277 | 129 | 133 | 158 | 141 | R1136b | 155 | 151 | 169 | 168 | 140 | 296 | 153 | 207 (CD) | x |
| 27 | N | 257 | 280 | 132 | 136 | 161 | 144 | 1137 | 158 | 154 | 172 | 171 | 143 | 299 | 156 | 210 (CG) | 223 (CG) |
| 28 | h | L258 | L281 | L133 | F137 | V162 | I145 | C1138 | L159 | I155 | I173 | L172 | I144 | V300 | I157 | I211 (CG1) | x |
| 29 | h | L259 | M282 | L134 | L138 | M163 | M146 | M1139 | A160 | L156 | L174 | L173 | M145 | L301 | L158 | L212 (CG) | x |
| 30 | h | L260 | L283 | I135 | I139 | I164 | L147 | V1140 | V161 | L157 | I175 | I174 | I146 | M302 | L159 | L213 (CG) | x |
| 31 | l | V266 | I289 | I141 | I150 | L171 | I157 | V1146 | L167 | I163 | L182 | I180 | V152 | I546 | V165 | I219 (CG1) | x |
| 32 | K | 267 | 290 | 142 | Y151p | R172b | 158 | 1147 | 168 | 164 | 183 | Q181p | 153 | 547 | 166 | Q220p (CD) | x |
| 33 | l | I268 | I291 | L143 | V152 | L173 | I159 | I1148 | I169 | L165 | L184 | V182 | V154 | I548 | L167 | I221 (CG1) | 235 (CG) |
| 34 | D | 270 | 293 | 145 | 154 | 175 | 161 | 1150 | 171 | 167 | 186 | 184 | 156 | 550 | 169 | 223 (CG) | x |
| 35 | F | 271 | 294 | 146 | 155 | W176r | 162 | 1151 | 172 | 168 | 187 | 185 | 157 | L551h | 170 | 224 (CG) | x |
| 36 | G | 272 | 295 | 147 | 156 | 177 | 163 | 1152 | 173 | 169 | 188 | 186 | 158 | 552 | 171 | 225 (CA) | x |
| 37 | T | P293s | 313 | 165 | 181 | S194d | 180 | x | 188 | 186 | 204 | 201 | x | 567 | 185 | 245 (CB) | x |
| 38 | r | W296 | Y316 | Y168 | Y184 | F197 | F183 | W1175 | Y191 | Y189 | Y207 | Y204 | Y182 | Y570 | W188 | Y248 (CD2) | x |
| 39 | s | A298 | A318 | A170 | S186 | G199 | A185 | A1177 | A193 | A191 | P209 | A206 | S184 | S572 | A190 | S250 (CB) | x |
| 40 | P | 299 | 319 | 171 | I187h | 200 | 186 | 1178 | 194 | 192 | 210 | 207 | 185 | 573 | 191 | 251 (CG) | x |
| 41 | E | 300 | 320 | 172 | N188p | 201 | 187 | 1179 | 195 | 193 | 211 | 208 | 186 | 574 | 192 | 252 (CD) | x |
| 42 | D | 312 | 332 | 185 | 200 | 214 | 199 | 1191 | 208 | 211 | x | 220 | 198 | 586 | 207 | 264 (CG) | x |
| 43 | W | 314 | 334 | 187 | x | 216 | 201 | 1193 | 210 | 213 | 226 | 222 | Y200r | 588 | 209 | 266 (CD2) | x |
| 44 | S | x | G335v | 188 | A203v | 217 | 202 | 1194 | 211 | 214 | 227 | A223v | 201 | 589 | 210 | A267s (CB) | x |
| 45 | G | 317 | 337 | 190 | 205 | 219 | 204 | 1196 | 213 | 216 | 229 | 225 | 203 | A591v | 212 | 269 (CA) | x |
| 46 | h | M323 | M343 | M196 | F211 | M225 | L210 | I1202 | M219 | L222 | M235 | M231 | V209 | L597 | L218 | L275 (CG) | x |
| 47 | F | x | 350 | 203 | x | 233 | 217 | x | 226 | 229 | 242 | 238 | 216 | 604 | x | 282 (CG) | x |
| 48 | h | C367 | L384 | M266 | Y256 | L304 | L255 | C1245 | M291 | F267 | C270 | L272 | A254 | M686 | C261 | L316 (CG) | x |
| 49 | L | W368h | 385 | 267 | M257h | 305 | 256 | W1246h | 292 | 268 | 271 | 273 | 255 | 687 | 262 | 317 (CG) | x |
| 50 | P | 372 | 389 | 271 | x | x | 260 | 1250 | A296s | 272 | 275 | L277h | 259 | 691 | 266 | A321s (CB) | x |
| 51 | R | 375 | 392 | 274 | x | 312 | 263 | 1253 | 299 | 275 | 278 | 280 | 262 | 694 | 269 | 324 (NE) | x |
| 52 | H | x | 406 | 283 | x | 321 | 272 | x | 308 | 284 | 287 | 294 | D272c | 703 | 278 | 339 (CG) | x |

^*^Residues (and atoms) used in constraint based modeling.

The amino acid or amino-acid category of the conserved residue is listed under type. For each structure the residue identifier corresponding to the conserved point is indicated (listed as x if the point is absent). For category types the amino acid present in each structure is indicated before the identifier. If a sample is missing a conserved amino acid but has a similar residue in the same location then the shared category is listed after the identifier. a, acidic; l, aliphatic; r, aromatic; b, basic; c, charged; h, hydrophobic; p, polar; s, small; v, very small.
